# Critical structural elements for the antigenicity of wheat allergen LTP1 (Tri a 14) revealed by site-directed mutagenesis

**DOI:** 10.1038/s41598-022-15811-5

**Published:** 2022-07-18

**Authors:** Hamza Mameri, Jean-Charles Gaudin, Virginie Lollier, Olivier Tranquet, Chantal Brossard, Manon Pietri, Didier Marion, Fanny Codreanu-Morel, Etienne Beaudouin, Frank Wien, Yann Gohon, Pierre Briozzo, Sandra Denery-Papini

**Affiliations:** 1 INRAE, UR 1268 Biopolymères Interactions Assemblages (BIA), 44316 Nantes, France; 2 INRAE, UR BIA, 44316 Nantes, France; 3 INRAE, PROBE Research Infrastructure, BIBS Facility, 44316 Nantes, France; 4 CHU Luxembourg, Centre Hospitalier de Luxembourg, Kanner Klinik, 1210 Luxembourg, Luxembourg; 5 Service d’Allergologie, Hôpital de Mercy, CHR Metz, 57000 Metz, France; 6 Synchrotron Soleil, Saint-Aubin, 91192 Gif-sur-Yvette, France; 7 INRAE, UMR 1318 Institut Jean-Pierre Bourgin, 78026 Versailles, France; 8 Present Address: UMR 1208 IATE, Univ Montpellier, INRAE, L’Institut-Agro Montpellier, 34060 Montpellier, France; 9 Present Address: INRAE, UMR 0588 Biologie intégrée pour la valorisation de la diversité des arbres et de la forêt (BIOFORA), 45075 Orléans, France; 10 Present Address: INRAE UMR 1163 Biodiversité et Biotechnologie Fongiques (BBF), 13288 Marseille, France; 11 Present Address: Institut de Cancérologie de l’Ouest, Centre René Gauducheau, 44805 Saint Herblain Cedex, France

**Keywords:** Immunological disorders, Immunochemistry, Protein folding

## Abstract

Lipid transfer proteins (LTPs) were identified as allergens in a large variety of pollens and foods, including cereals. LTPs belong to the prolamin superfamily and display an α-helical fold, with a bundle of four α-helices held together by four disulfide bonds. Wheat LTP1 is involved in allergic reactions to food. To identify critical structural elements of antibody binding to wheat LTP1, we used site-directed mutagenesis on wheat recombinant LTP1 to target: (i) sequence conservation and/or structure flexibility or (ii) each disulfide bond. We evaluated the modifications induced by these mutations on LTP1 secondary structure by synchrotron radiation circular dichroism and on its antigenicity with patient’s sera and with mouse monoclonal antibodies. Disruption of the C28–C73 disulfide bond significantly affected IgE-binding and caused protein denaturation, while removing C13–C27 bond decreased LTP1 antigenicity and slightly modified LTP1 overall folding. In addition, we showed Lys72 to be a key residue; the K72A mutation did not affect global folding but modified the local 3D structure of LTP1 and strongly reduced IgE-binding. This work revealed a cluster of residues (C13, C27, C28, C73 and K72), four of which embedded in disulfide bonds, which play a critical role in LTP1 antigenicity.

## Introduction

Lipid transfer proteins (LTPs), also known as non-specific lipid transfer proteins (ns-LTPs), form a widespread family of plant proteins characterized by a hydrophobic cavity that allows binding with a large variety of lipids^1^. In plants, LTPs are associated with different events and signalling pathways related to development and defence against pathogens and environmental stress^2^. Although their precise function(s) is(are) still largely unknown, recent findings indicate that they are at least involved in lipid transfer between organelles^3,4^. The presence of lipid ligands is also a frequent characteristic of plant allergens and appears relevant to allergic sensitization events^5^. Plant LTPs belong to the prolamin protein superfamily, which includes several important types of allergens related to legumes, tree nuts, cereals, fruits and vegetables^6,7^. The LTP family comprises two structurally related types, LTP1 and LTP2^8^. Both are small globular proteins with *M*_r_ ≈ 9–13 kDa, and a basic pI (9–10). LTP1 and LTP2 display low sequence identities but a similar α-helical fold, with a bundle of four α-helices numbered H1–H4 held together by four disulfide bonds, allowing formation of a hydrophobic cavity^9^. The 3D structure of wheat LTP1 has been resolved by nuclear magnetic resonance (NMR) (PDB ID 1GH1)^10^ and X-ray crystallography (PDB ID 1BWO)^11^. The protein contains a skeleton of eight cysteine residues forming four disulfide bonds that are located at positions C3–C50, C13–C27, C28–C73 and C48–C87.

LTPs, especially LTP1, have been identified as pollen respiratory allergens, such as in mugwort (Art v 3)^12^, as well as plant-food allergens in a large variety of foods, particularly in *Rosaceae* fruits and in their *Prunoideae* subfamily^13–16^.

LTP allergy syndrome, initially described for older children and adults in Spain and Italy, is characterized by multiple plant-food sensitizations and potentially severe clinical reactions in patients sensitized to LTP1. Peach is the most frequent offending food for these patients, and immunoglobulin E (IgE) specific for peach LTP1 Pru p 3 is detected in a very high percentage of patients (75–100% according to studies^17–19^). Although less prevalent than fruits, cereals—notably maize and wheat^20,21^—may also be involved in the cross-reaction between different LTP sources. In this context, wheat LTP1 Tri a 14 was shown to be involved in severe reactions such as anaphylaxis and wheat-dependent exercise-induced anaphylaxis^22^.

Apart from its role in LTP syndrome, wheat LTP1 has been shown to be an important allergen in food allergy to wheat^23,24^. About 33–35% of children allergic to wheat had IgE specific for LTP1^25^. In addition, sensitization to Tri a 14 is observed in baker’s asthma^20,26–28^.

Identification of epitopes on plant LTPs has focused on understanding the molecular mechanisms of sensitization to these pan-allergens frequently involved in cross-reactions. Findings would be useful in the context of allergen immunotherapy that aims at developing safe and effective treatment of food allergies using peptides, variants or modified proteins^29–33^. We previously studied epitope mapping using immobilized synthetic peptides covering amino acid sequences of wheat LTP1 (Tri a 14) and sera from children allergic to wheat in foods. We found that only a few sequential epitopes are recognized by IgE from only a few patients^34^, suggesting that most LTP1 B-cell epitopes are conformational. This is probably a consequence of the high thermal stability of this protein during food processing and its resistance to gastrointestinal digestion, allowing sensitization^35–37^. Identifying conformational epitopes requires time-consuming and extensive strategies. The most common technique for locating and mapping conformational epitopes is directed mutagenesis of the protein allergen. A complementary approach, known as mimotope mapping, uses random peptide libraries that mimic epitopes. Both techniques have been applied successfully to identify IgE-binding and T-cell epitopes of peach LTP Pru p 3^38–40^. The mimotope approach has also revealed a wheat LTP1 conformational epitope for IgE from patients with baker’s asthma^35^ and its probable contribution to cross-reactivity between Pru p 3 and Tri a 14. Recently, conformational epitopes were mapped on the mugwort pollen allergen Art v 3 using directed mutagenesis in combination with a novel approach, H/D (hydrogen/deuterium) exchange memory NMR. This method measures differences in signals between the allergen–antibody complex and free allergen^41^.

**Figure 1:**
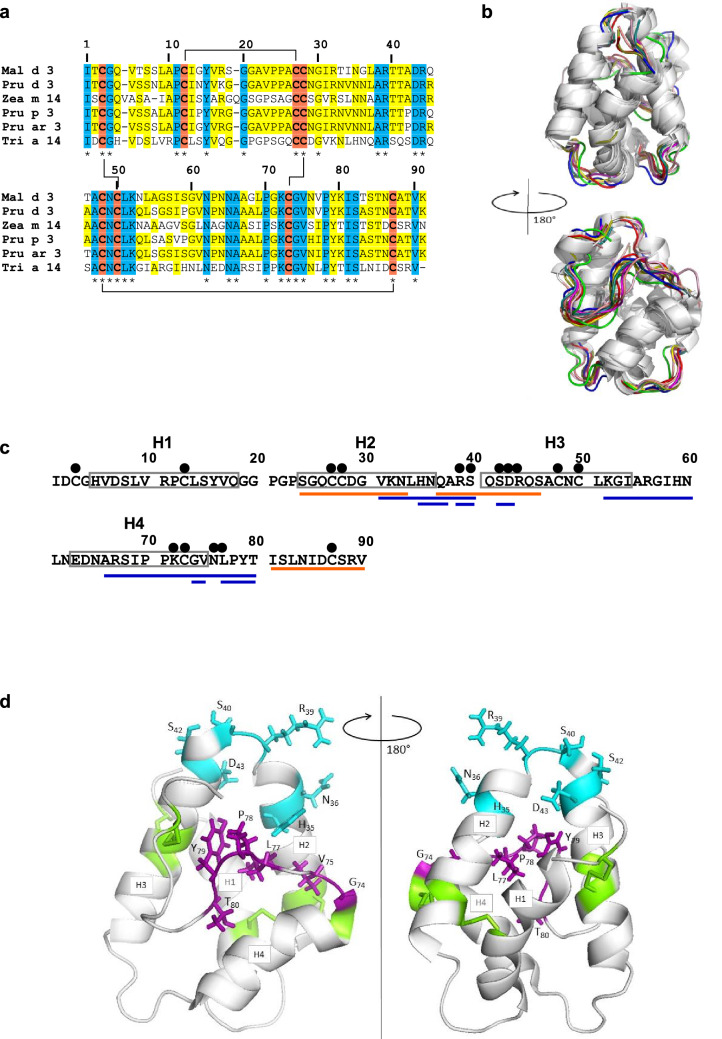
Wheat LTP1 epitopes. (**a**) Alignment of LTP1s from different plant species. Mal d 3 (*Malus domestica*, UniProtKB–B6CQU3), Pru d 3 (*Prunus domestica*, UniProtKB–P82534), Pru p 3 (UniProtKB–P81402), Zea m 14 (*Zea mays*, UniProtKB–A0A317Y1E3), Pru ar 3 (*Prunus armeniaca*, UniProtKB–P81651), Tri a 14 (*Triticum aestivum* UniProtKB–P24296) were aligned with Clustal Omega (https://www.ebi.ac.uk/Tools/msa/clustalo/). Location and pairing connection of conserved disulfide bonds (–) are in bold and orange, amino acid residues conserved in at least three sequences are in yellow, and residues strictly conserved in all six sequences are in cyan and indicated with *. (**b**) Superposition of nine plant LTP1 3D structures. The structures shown are those from wheat (1GH1.PDB, red), maize (1AFH.PDB, green), mung bean (1SIY.PDB, blue), barley (3GSH.PDB, yellow), rice (1UVA.PDB, pink), eggplant (5TVI.PDB, orange), hazelnut (4UXW.PDB, brown), mugwort (6FRR.PDB, light pink) and peach (2B5S.PDB turquoise). (**c**) Location of epitopes on wheat LTP1 sequence. Sequential IgE epitopes described by Denery-Papini et al*.*^34^ in food allergy to wheat are underlined in orange. Sequential and conformational epitopes described by Tordesillas et al.^35^ in baker’s asthma are underlined in blue. The position of mutations is indicated (filled black circle). (**d**) Location of conformational epitopes and disulfide bonds on wheat LTP1 3D structure. LTP1 (PDB accession no. 1BW0a) 3D structure (ribbon representation) contains four α-helix (H1–H4) and four disulfide bonds (in green). Amino acid residues (with side chains) of the two parts of the conformational epitope described in the case of baker’s asthma by Tordesillas et al*.*^35^ are in blue and violet. LTP: lipid transfer protein. PDB: Protein Data Bank.

To identify critical structural elements of antibody binding to wheat LTP1 in patients suffering from food allergy to wheat, we used site-directed mutagenesis on wheat recombinant LTP1. Disulphide bonds, which are conserved among plant LTP1s, have been shown to be involved in stabilizing the 3D structure and to be crucial for IgE/LTP1 interactions. We previously showed that reduction/alkylation of the four disulfide bonds of wheat LTP1 abolished IgE-binding in the context of food allergy to wheat^34^. A large set of protein variants was produced to evaluate the role of each disulfide bond and how some amino acid residues contribute to IgE/LTP1 interactions. Mutations were notably designed to evaluate the importance of the conservation of primary amino acid sequences among several plant-food LTPs and of their location in the 3D structure as key elements for IgE-binding. Indeed, despite mild conservation in primary structure (Fig. 1), plant LTP1s exhibit remarkable conservation of their 3D structure, while least constrained parts of the structure, like unstructured loops, contribute to the flexibility that allows lipid positioning in LTP cavity. Mutations induced on conserved/unconserved residues may elucidate allergen cross-reaction or co-sensitization events, while changes in spatially flexible residues explore the effect of molecular plasticity on antibody interactions.

We evaluated the modifications induced by these mutations on LTP1 antigenicity with sera from patients suffering from food allergy to wheat and with mouse monoclonal antibodies (mAbs) directed against natural and denatured LTP1. Modification of LTP1 triggering capacity was also assessed by rat basophilic leukaemia (RBL) cell degranulation assay. Finally, structures of wheat recombinant LTP1 and mutant LTP1s (Mut-LTPs) were analysed by SRCD (synchrotron radiation circular dichroism) and by computational analysis for one hypoallergenic variant.

## Results

### Rational design of mutations

#### Mutations targeting sequence conservation and protein flexibility

We selected residues R39, S40, S42, D43 and R44 for mutations because some of these residues are relatively conserved among plant LTP1s (R39, D43, R44) (Fig. 1a and Supplementary Fig. S1a), or because they belong to a short loop and accessible area (R39, S40, S42) (Fig. 1d and Supplementary Fig. S1b). Moreover, these residues are included in the IgE sequential epitope _37_QARSQSDRQS_46_, which was previously recognized by both patients with allergy to food and wheat LTP1-sensitized mice^34^. Some of these residues are also present in the conformational epitope (H_35_N_36_R_39_S_40_S_42_D_43_) recognized by a pool of sera from Spanish patients with baker’s asthma, co-sensitized with peach and wheat LTPs^35^ (Fig. 1c,d). Accordingly, six variants with single or double mutations or insertions (R39A, R39-AAA-S40, S40G-S42G, D43N and D43E-R44K, R39A-D43N) were designed and produced as recombinant proteins (Table 1).

**Table 1:** Summary of mutations and their theoretical effect on structure.

Target	Protein/variant name	Theoretical structural effect	Sequence conservation	Structure flexibility	Disulfide bond
Whole protein	Ra-LTP	Unfolded structure	–	–	Yes
	Wt-LTP	Effect of heterologous expression as Trx fusion protein	–	–	–
S-S bond	C3S/C50S	Partial unfolding			Yes
	C13S/C27S				Yes
	C28S/C73S				Yes
	C48S/C87S				Yes
S40 & S42	S40G-S42G	Polar → aliphatic area	Yes	Yes	
R39	R39A	No H exchange, contact surface reduced	Yes	Yes	
	R39-AAA-S40	Local surface alteration	–	Yes	–
	R39A-D43N	H exchange alteration	Yes	Yes	
K72	K72A	No H exchange, contact surface reduced	Yes	–	–
N76	N76A	No H exchange, contact surface reduced	–	Yes	–
	N76-AAA-L77	Local surface alteration	–	Yes	–
D43	D43N	Charge inversion	Yes	–	–
	D43E-R44K	Contact surface slightly increased	Yes	–	–

LTP: lipid transfer protein. Ra-LTP: reduced/alkylated LTP1. S-S: disulfide. Trx: thioredoxin. Wt-LTP: recombinant wild-type wheat LTP1.

Three other residues (K72, N76, L77) located at the end of α-helix 4 and in the flexible C-terminal domain of LTP1 were targeted as well to design three additional variants within the first mutation set. N76 and K72 have relatively high accessibility in the LTP1 3D structure, with K72 being a highly conserved residue among plant LTP1s (Fig. 1 and Supplementary Fig. S1, Table 1). K72, N76 and L77 are in the vicinity of the amino acid residue set G_74_V_75_L_77_P_78_Y_79_T_80_ from the conformational epitope described for patients with baker’s asthma in Tordesillas et al*.*^35^. The introduction of three alanine between the N76 and L77 or R39 and S40 residues was done to increase the loop size between H2-H3 helices and H3-H4 helices, respectively.

#### Mutations targeting disulfide bonds

Accordingly, to study the contribution of each disulfide bond, we designed a second set of four variants (C3S-C50S, C13S-C27S, C28S-C73S and C48S-C87S) by removing one bond at a time (Table 1).

### Recombinant LTP1 production and purification

Recombinant wild-type wheat LTP1 (Wt-LTP) and the series of variants were expressed in *Escherichia coli* Origami B strain as fusion proteins with N-terminal thioredoxin (Trx) in order to produce them in a soluble and well-folded form^42^. Only proteins produced in the soluble fraction after bacterial lysis were submitted to purification by Ni^2+^-affinity chromatography followed by anion-exchange chromatography. The purity of recombinant proteins was checked by 15% SDS-PAGE and western blot (not shown). A few hundred micrograms of each mutant were obtained and used for subsequent experiments.

### IgE-reactivity to the Wt-LTP

**Figure 2:**
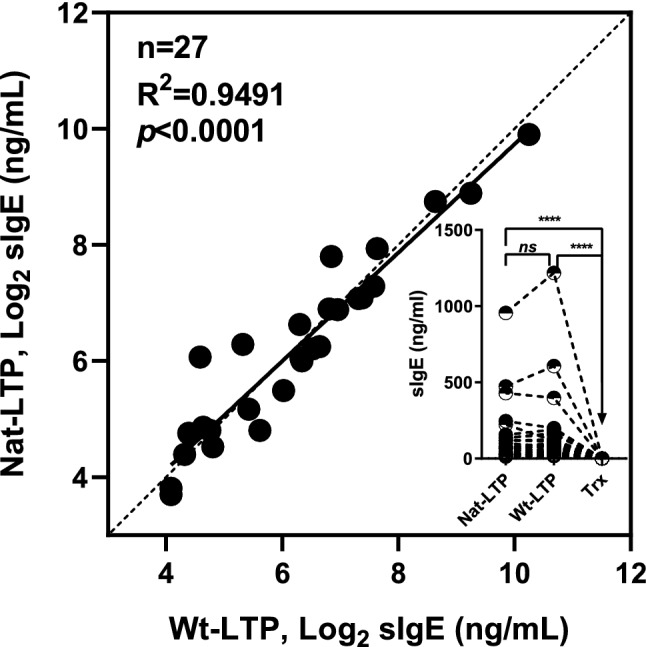
IgE-binding to Nat-LTP and Wt-LTP. Pearson correlation between IgE-binding on Nat-LTP and Wt-LTP measured by ELISA (large panel). Comparison of IgE-binding with Nat-LTP, Wt-LTP and Trx (small panel). Each dot corresponds to a patient. ELISA: enzyme-linked immunosorbent assay. Nat-LTP: natural lipid transfer protein. Trx: thioredoxin. Wt-LTP: recombinant wild-type wheat LTP1.

Sera from 27 patients with food allergy to wheat and sensitized to wheat LTP1 (Table S1) were used to compare IgE reactivity towards natural LTP1 (Nat-LTP) and recombinant Wt-LTP. Trx was included as a control. No Trx-specific IgE was detected in patient sera. Pearson correlation analysis showed a highly significant correlation between Nat-LTP and Wt-LTP (R^2^ = 0.9491, *p* < 0.0001, *n* = 27; Fig. 2, large panel). Dunn’s multiple comparisons test indicated no significant difference in IgE-binding to Nat-LTP and Wt-LTP (rank sum differences = − 5, *p* > 0.9999, *n* = 27) (Fig. 2, small panel). Seventeen (17) of these sera with sufficient volumes were used in subsequent experiments.

### IgE reactivity of mutant LTPs and induced RBL cell degranulation

**Figure 3:**
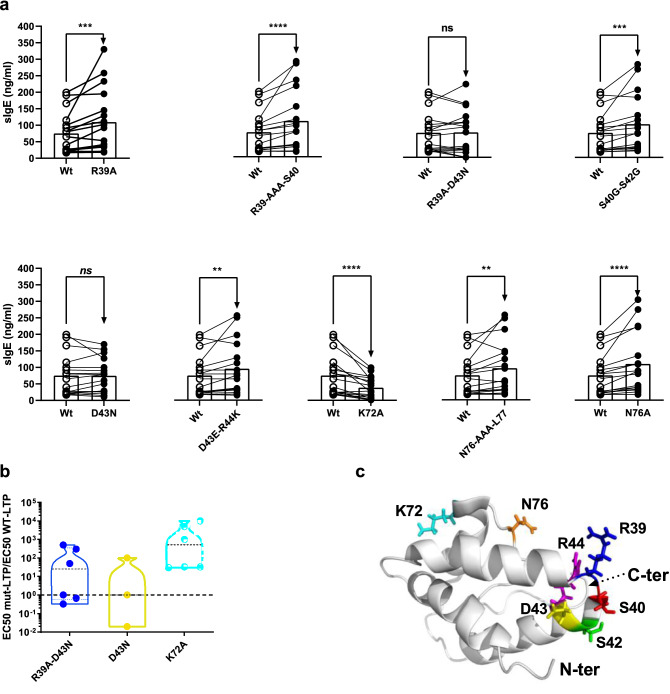
IgE-binding to LTP1 variants affecting sequence conservation or protein flexibility and RBL degranulation ability assay. (**a**) Comparison of IgE-binding to Wt-LTP and LTP1 variants measured by ELISA. Medians are considered statistically different if *p* < 0.05, Wilcoxon signed rank test, *n* = 17. ns: not significant. (**b**) RBL degranulation ability of R39-D43N, D43N and K72A variants. The values are expressed as ratios of EC_50_ of Mut-LTP to that of Wt-LTP. The dashed horizontal bar denotes the value of 1, indicating that EC_50_ of Wt-LTP and Mut-LTP are equal. (**c**) The position of the mutated residues (shown in sticks) is highlighted in different colours on the LTP1 3D structure (1GH1.PDB). EC_50_: half maximal concentration; ELISA: enzyme-linked immunosorbent assay. LTP1: lipid transfer protein. Mut-LTP: mutant LTP1. RBL: rat basophilic leukaemia. Wt-LTP: recombinant wild-type wheat LTP1.

**Figure 4:**
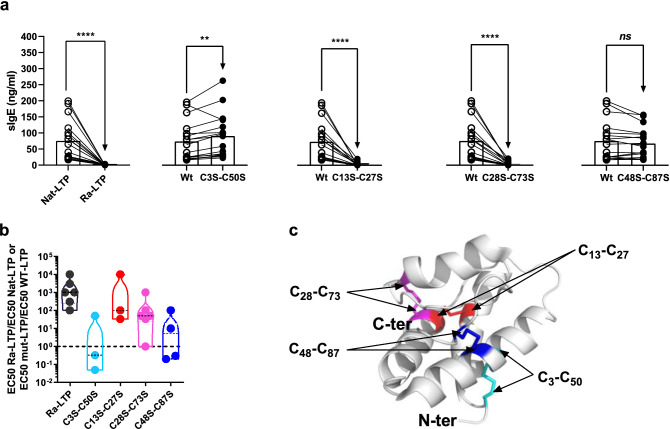
IgE-binding to reduced alkylated and disulfide bond variants and RBL degranulation ability assay. (**a**) Comparison of IgE-binding to Nat-LTP and Ra-LTP and to Wt-LTP and LTP1 bisulfide bond variants measured by ELISA. Medians are considered statistically different if *p* < 0.05 (Wilcoxon signed rank test, *n* = 17). (**b**) RBL degranulation ability of Ra-LTP and Mut-LTP (disulfide bond variants). The values are expressed as ratios of EC_50_ of Ra-LTP to that of Nat-LTP and of EC_50_ of Mut-LTP to that of Wt-LTP. The dashed horizontal bar denotes the value of 1, indicating that EC_50_ of Wt-LTP and Mut-LTP are equal. **c**. Position of the cysteine residues is highlighted in different colours on the LTP1 3D structure (1GH1.PDB). EC_50_: half maximal effective concentration. ELISA: enzyme-linked immunosorbent assay. LTP: lipid transfer protein. Mut-LTP: mutant LTP1. ns: not significant. Ra-LTP: reduced/alkylated LTP1. RBL: rat basophilic leukaemia. Wt-LTP: recombinant wild-type wheat LTP1.

These 17 sera were used in fluorimetric enzyme-linked immunosorbent assay (F-ELISA) to measure concentrations in specific IgE for Wt-LTP, reduced/alkylated LTP1 (Ra-LTP) and 13 Mut-LTPs. For better clarity, we separately analysed the variants affecting sequence conservation and structure flexibility with either single or double mutations (Fig. 3) and those targeting disulfide bonds (Fig. 4).

### Variants related to sequence conservation and protein flexibility

The introduced mutations had a significant effect on IgE-binding capacity compared with Wt-LTP, except for the R39A-D43N and D43N variants, which showed no difference as regards to specific IgE concentration median values (Fig. 3a). Nevertheless, a few individual sera displayed decreased IgE-binding for these variants compared with Wt-LTP, notably for the double mutation R39A-D43N. The R39A, R39-AAA-S40, S40G-S42G, D43E-R44K, N76A and N76-AAA-L77 variants induced significantly stronger IgE-binding, with a median value increase ranging from + 5 to + 19 ng mL^–1^, whereas the K72A mutant showed a dramatic and systematic decrease in IgE-binding (–20 ng mL^–1^ decrease in median value).

To confirm our results at the cellular level, in a system that mimics in vivo triggering, we assayed variants inducing a significant reduction in IgE-binding (K72A) or no effect (D43N and R39A-D43N) for their ability to induce basophil degranulation (RBL cells), with three to six sera (Fig. 3b). No degranulation was observed with Trx alone, whatever the sera (data not shown). Wt-LTP displayed very slightly lower biological activity than Nat-LTP in this cell model. We measured the half-maximal eliciting concentration (EC_50_) in the range < 0.1–10 ng mL^–1^ and 0.1–30 ng mL^–1^ for Nat-LTP and Wt-LTP, respectively (not shown). For variants, EC_50_ values were compared with those obtained with Wt-LTP. For the K72A mutant, a 30-fold increase in EC_50_ was observed for three sera, while for three other sera the EC_50_ increase was in the range of 10^3^–10^4^, which indicates markedly reduced eliciting potency. The two other mutations (D43N, R39A-D43N) did not change, increased or decreased (increase in EC_50_ by a factor of 50–500) the ability of proteins to induce degranulation, depending on the sera. Even when performed with a limited number of sera, these results confirmed the ELISA results and identified the K72A mutant as the one with the greatest effect on LTP1 antigenicity.

### Disulfide bond variants

To better understand the contribution of disulfide bonds to wheat LTP1 IgE reactivity, we measured IgE-binding to Nat-LTP, to the same protein after reduction and alkylation (Ra-LTP) and to recombinant disulfide bond variants of LTP1s by ELISA. The results presented in Fig. 4a show that, as expected, reduction of all Nat-LTP disulfide bonds dramatically affected specific IgE-binding. Mutation of either the C3-C50 or C48-C87 disulfide bond increased or left unchanged medians of specific IgE concentrations, and led to a slight decrease in IgE-binding for only one serum and three sera, respectively. In contrast, mutation of either the C13-C27 or C28-C73 disulfide bond resulted in a highly significant drop in IgE-binding capacity (− 60 and − 64 decrease in median value, respectively, with *p* < 0.0001). All sera tested showed very low or no binding at all to these two variants (83–100% reduction in specific IgE concentrations compared with Wt-LTP).

We compared the RBL degranulation capacity of Wt-LTP and of the different disulfide bond variants, using three to six sera (Fig. 4b). We also compared Nat-LTP- and Ra-LTP-induced RBL degranulation as a control. For all sera, reduction of Nat-LTP strongly affected its triggering potency, since EC_50_ measured for Ra-LTP was between 100 and 10^4^ times higher than that measured for Nat-LTP. Significant increases in EC_50_ were also observed with the C13S-C27S and C28S-C73S variants compared with Wt-LTP (for five out of six sera, EC_50_ increased from 30 to 10^3^, and for three out of three sera, between 30 and 10^4^, respectively). For the two other variants (C3S-C50S and C48S-C87S), mutations increased, let unchanged or decreased (increase up to 50 or 100 the EC_50_ ratios) the ability to induce degranulation, depending on the sera. These results confirm the ELISA results and show the important role played by the C13-C27 and C28-C73 disulfide bonds in wheat LTP1 antigenicity and triggering activity.

### Reactivity of mAbs directed against natural and chemically modified LTPs

To evaluate the effect of 3D structure on LTP1 antigenicity, mice were immunized either with Nat-LTP or Ra-LTP. Monoclonal antibodies were obtained and characterized in ELISA for their reactivity towards the immunogens and Wt-LTP, as well as towards variants selected for their reduced IgE-binding capacity. The following variants (C13S-C27S and K72A) and (C3S-C50S, C48S-C87S, R39A-D43N, D43N) were used for their strong or slight effect on IgE binding respectively. The C28S-C73S LTP1 variant was not available for this analysis. From mice immunized with Nat-LTP, 14 clones secreting mAbs that bound to this LTP were obtained; none of them recognized Ra-LTP (Table 2). Six of the mAbs did not react with Wt-LTP or with any of the tested variants, the eight other mAbs bound equally to Nat-LTP and Wt-LTP. They also bound to C3S-C50S, C48S-C87S, D43N and R39A-D43N variants, with the exception of two mAbs that bound neither to C48S-C87S nor to R39A-D43N variants. None of the mAbs bound to C13S-C27S or K72A variants, apart from one mAb that reacted slightly to the K72A mutant.

**Table 2:**
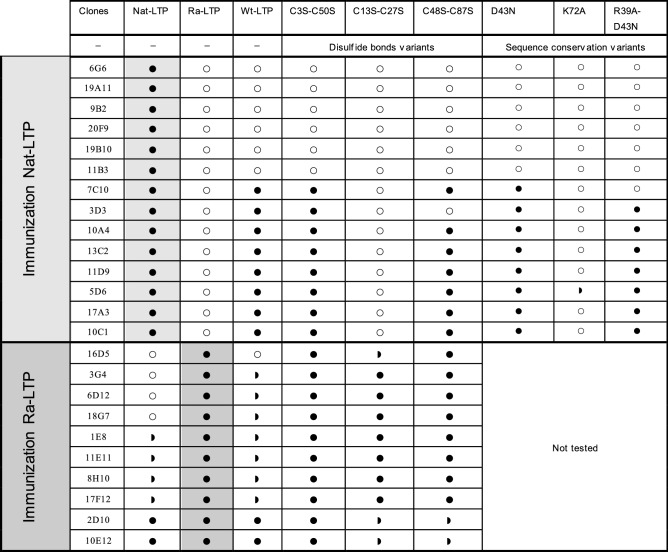
Monoclonal mouse antibody reactivity.

Reactivity of monoclonal antibodies obtained by immunization with LTP1 in its native form (Nat-LTP) or after reduction/alkylation (Ra-LTP). Reactivity against Nat-LTP, Ra-LTP or the tested Mut-LTP variants was found to be equivalent (●), reduced but positive () or null (○) when compared with reactivity against the immunogen. LTP: lipid transfer protein. Ra-LTP: reduced/alkylated LTP1.

Ten clones secreting mAbs that bound to Ra-LTP were obtained from mice immunized with this protein. All the mAbs were able to bind to the three variants lacking disulfide bonds (C3S-C50S, C13S-C27S and C48S-C87S). Six mAbs also reacted with Wt-LTP and Nat-LTP, two of them with equal intensity; four other mAbs showed less reactivity compared with Ra-LTP. Four mAbs from this immunization did not bind to Nat-LTP. Nonetheless, three of them displayed a limited but significant response to Wt-LTP.

### Structural integrity and surface topography

**Figure 5:**
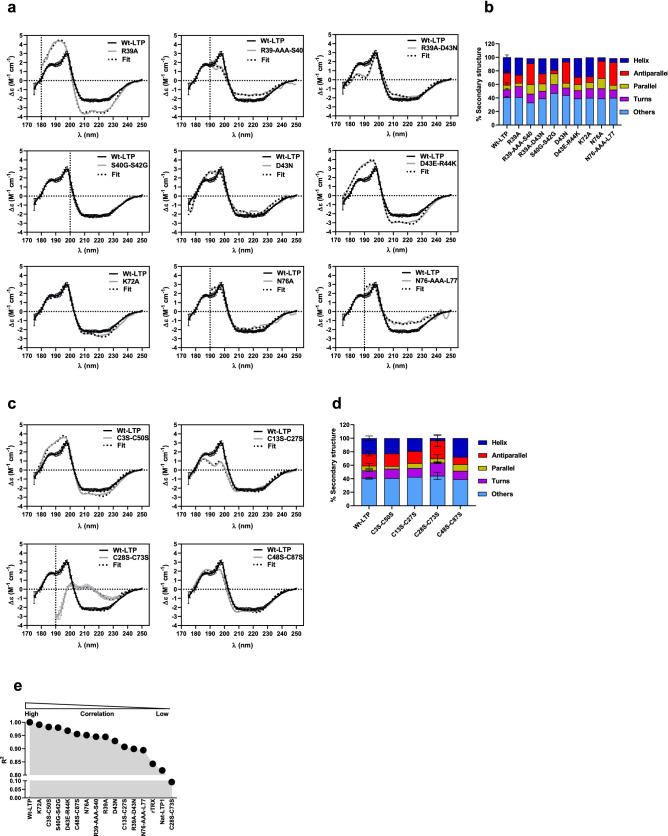
SRCD of LTP1s. SRCD spectra and secondary structure content estimation of wild-type and LTP1 variant proteins. The fit corresponds to the theoretical curve used for secondary structure calculation. (**a**) Spectra for variants affecting sequence conservation or protein flexibility. (**b**) Corresponding bar plot indicating secondary structure content. (**c**) Spectra for variants affecting disulfide bonds. (**d**) Corresponding bar plot indicating secondary structure content. (**e**) Pearson correlation between the Wt-LTP CD signal and those of the different LTP1 variants. The variants are classified according to the square of the correlation coefficient (R^2^) from highest to lowest. LTP: lipid transfer protein. SRCD: synchrotron radiation circular dichroism. Wt-LTP: recombinant wild-type wheat LTP1.

To relate the effect of introduced mutations on antibody binding (human IgE and mouse mAbs) and degranulation events to changes in protein folding, we analysed the selected variants using SRCD (Fig. 5). Wt-LTP, Nat-LTP and recombinant Trx spectra were recorded as references (Fig. S2). As expected, the CD spectrum of Nat-LTP showed a typical CD signal for α-helical structures, with a maximum at 192 nm and a double minimum at 208–210 nm and 223–224 nm. Trx spectra displayed a negative dichroic peak centred on 220 nm, indicating the presence of β-sheet structures, as well as a positive peak at 197 nm consistent with its known α–β structure. The Wt-LTP signal displayed a maximum at 197 nm found for Trx and a shoulder around 185–190 nm consistent with LTP1 (Fig. S2).

### Variants related to sequence conservation and protein flexibility

The CD spectra of LTP1 variants targeting sequence conservation and/or flexibility presented in Fig. 5a are fairly close to that of Wt-LTP, with the exception of the R39A mutant spectrum. All the variants displayed features from both Trx and LTP1 proteins, notably the positive peak at 197 nm and the shoulder around 185–190 nm. Some spectra were cut off at the indicated wavelength because low quality of the CD signals made it difficult to draw firm conclusions. The spectra of some proteins such as S40G-S42G and D43N and N76A are very close, which suggests that their overall secondary structure is similar. For some other variants, more marked differences were observed in signal intensities at the positive peaks, particularly at the shoulder around 185–190 nm for R39A-D43N sample, meaning that mutations induced structural changes in LTP1.

Estimated changes to secondary structures are reported in Fig. 5b. Wt-LTP includes helices (23%), parallel and antiparallel β-sheets (7% and 18%, respectively), turns (11%) and other structures (40%). R39-AAA-S40, D43N, N76A and N76-AAA-L77 mutations induced a dramatic decrease in α-helical structures (− 16 to − 18%) and a clear increase in parallel (+ 8% to 16%) and antiparallel (+ 1% to + 9%) β-sheet structures, whereas the other structures are less affected by mutations.

The S40G-S42G double mutation induced a different effect, with a decrease in α-helices (− 6%) and antiparallel β-sheets (− 14%) and an increase in parallel (+ 10%) β-sheet structures and other structures (+ 7%). The turn structures remained stable. R39A, D43E-R44K and K72A variants did not show large variations in their percent of secondary structures.

### Disulfide bonds variants

As shown in Fig. 5c, the CD spectra of disulfide bonds variants are likely close to Wt-LTP1 except for the C28S-C73S mutant which has an atypical CD signal and showed a dramatic change in secondary structure.

Some small variations in the C13S-C27S mutant particularly at the shoulder around 185–190 nm could be observed.

Secondary structure estimation for the C28S-C73S mutant showed a drop in α-helix content (− 20%), a marked increase in turn (+ 8%) and antiparallel strands (+ 8%), and a moderate increase in unordered structures (+ 4%) (Fig. 5d). The C48S-C87S mutant showed a slight increase in α-helix and parallel strand secondary structures (+ 4% and + 3%, respectively), and a decrease in antiparallel strand structures (− 8%), while the unordered structures remained stable. The secondary structures of C3S-C50S and C13S-C27S variants remained stable and equivalent to the wild-type protein. Together, the results of the disulfide bond mutations indicate that overall protein folding is mildly (C48-C87) or greatly (C28-C73) impacted by mutation of disulfide bonds. The C28-C73 mutation is strongly involved in stabilizing LTP1 structures.

Given the high number of proteins studied, we computed the Pearson correlation between the Wt-LTP CD signal and those of the other proteins (Fig. 5e). The variants were subsequently ranked based on distance from the wild-type protein. As shown in Fig. 5e, the K72A variant was the closest followed by the C3S-C50S variant and the C28S-C73S variant was the most distant.

**Figure 6:**
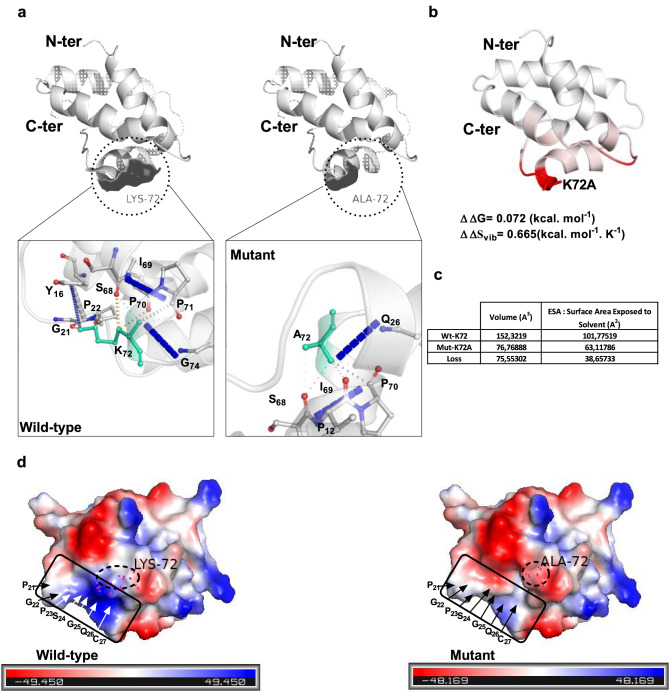
Interaction, surface and thermodynamic properties of the K72A LTP1 variant. (**a**) Wt-LTP and K72A variant 3D structures and interaction network predicted using the DynaMut web tool. The surface topology (upper panel) and the different residues affected by mutation are indicated (lower panel). (**b**) 3D structure showing the vibrational entropy of the K72A variant. Red indicates an increase in entropy. The predicted values of enthalpy (ΔΔG) and vibrational entropy (ΔΔS_vib_) appear below the 3D model. (**c**) Volumetric properties of Wt-LTP and K72A variants determined by the VLDP web server. The values and differences in volume and surface area exposed to solvent between wild-type and K72A variants are indicated. (**d**) Electrostatic properties of Wt-LTP and K72A variants were generated using PyMol. Red: negative charge; blue: positive charge. Boxes highlight the zone of major differences.

We then analysed the thermodynamics, surface topology and protein volume dimensions as well as the interatomic network of K or its A mutant residue at position 72 using the DynaMut^43^ and VLDP^44^ web servers, employing the 1GH1 PDB file or the K72A 3D structure model generated from DynaMut as templates (Fig. 6). The DynaMut analysis of the K72A mutation predicted stabilization of the structure with a very slightly dynamic structure based on a Gibbs free energy ΔΔG value of 0.072 kcal mol^–1^ and on a vibrational entropy (ΔΔS_vib_) of 0.665 kcal mol^–1^ K^–1^ (Fig. 6b). For residue 72, protein surface and volume analysis revealed a significant decrease in surface area exposed to solvent (–38.65 Å^2^) and volume (− 75.55 Å^3^) upon K72A mutation compared with the wild-type protein (Fig. 6c). Non-covalent interactions, including hydrogen bonds and van der Waals interactions, established between the K72 residue and other LTP1 residues decreased (as expected) and/or changed with Lys/Ala substitution from eight to five interactions. Interactions with neighbouring residues P70, I69 and S68 in the H4 helix were conserved, whereas those with residues positioned at the end of the H1 helix or in the loop between H1 and H2 (Y16, G21, P22), and with other residues in H4 (P71 and G74), were lost. Together, these in silico results indicate local structural modifications in the environment of position 72. Electrostatic analysis of Wt-LTP and K72A mutants using PyMOL software (Schrödinger, NY, USA) showed substantial modification in protein charge distribution on substitution of a positively charged residue by a hydrophobic residue (Fig. 6d). The mutation notably affected the residues located between P22 and C27 beyond the very nearby residues.

## Discussion

This study presents an attempt to use a large set of protein variants of wheat LTP1 to identify some of the key residues in its antigenicity and to expand our knowledge of epitope regions involved in food allergy to wheat.

Wt-LTP and Mut-LTPs were produced as recombinant fusion proteins with Trx and a spacer of 50 residues, including the His-tag. The impact of heterologous expression as a fusion protein and of mutations on the secondary structure of the protein was monitored using SRCD. Our structural data indicate that the two domains of the fusion protein (Trx and LTP1) may fold independently, in accordance with the presence of a rather long spacer. In addition, IgE-binding to Wt-LTP was comparable to that of Nat-LTP, indicating that Trx does not interfere with IgE interactions. This strategy has already been validated with Ara h 2, a major peanut allergen expressed in fusion with the carrier protein MBP^45^. Wt-LTP also displayed only slightly lower biological activity than Nat-LTP in a cell model of basophil degranulation.

Thirteen substitution variants of wheat LTP1 were produced in fusion with Trx and evaluated for IgE-binding with sera from patients allergic to wheat. Four of these variants (C3S-C50S, C48S-C87S, D43N and R39A-D43N) showed reduced binding by a limited number of sera, and two of them (C48S-C87S and R39A-D43N) also displayed loss of recognition by two anti-Nat-LTP mAbs. Three other variants (C13S-C27S, C28S-C73S and K72A) exhibited very significant reduction in IgE-binding for all or most sera. These mutations also markedly affected the protein’s triggering activity in the RBL assay. Similarly, variants C13S-C27S and K72A (C28S-C73S could not be evaluated) lost their antigenicity for a set of mAbs obtained by immunization with Nat-LTP, in contrast to Wt-LTP.

All other point or double mutations preserved IgE-binding. Surprisingly, several mutations (R39A, R39-AAA-S140, S40G-S42G, D43E-R44K, N76-AAA-L77 and N76A) caused a small but significant increase in LTP1 binding by IgE compared with Wt-LTP. The explanation for this phenomenon remains to be determined. Nevertheless, mutation may induce discrete changes, such as more favourable surface electrostatics and topology that may enhance interaction with IgE. The mutations might increase area sizes on mobile epitope residues. This seems to be the case for some LTP1 variants (R39-AAA-S40, N76-AAA-L77 and D43E-R44K). These mutations may also induce discrete changes elsewhere in protein structure, enabling easier accessibility. As an example, the presence of lipids could increase the accessibility of some flexible protein regions and was recently shown to impact the IgE-binding capacity of several plant LTPs^5,46,47^. In the case of wheat LTP1, it has been shown that the presence of lipid within the protein cavity increased its flexibility and accessibility to digestive enzymes, properties that may also influence its allergenic potential^37^.

The structural integrity and preservation of disulfide bonds is crucial for IgE-binding to wheat LTP1^34^ and has also been observed to be important for other LTPs^41,48^. Among the four LTP1 disulfide bonds that are characteristic of the prolamin superfamily, we identified two (C13-C27 and C28-C73) as critical elements for wheat LTP1 antigenicity. C28-C73 is also essential for protein self-assembly. Indeed, SRCD analysis showed that disruption of C28-C73, which links H2 and H4 helices, causes denaturation of the protein, with almost total disappearance of α-helix structures. A similar effect was previously observed after reduction/alkylation of all disulfide bonds^34^. Conversely, disruption of C13-C27, which links H1 and H2 helices, did not lead to protein denaturation. Rather, C13-C27 disruption induced only a small change in the SRCD spectra compared with Wt-LTP, indicating slight modification of overall folding, whereas IgE-binding and triggering capacity were notably reduced. These two disulfide bonds include the double cysteine motif (C27-C28) and hold H1, H2 and H4 helices together. By doing so, they probably serve as scaffolding and help in gathering the epitope residues. Yet some of these cysteine residues may also be directly involved in interaction of LTP1 with IgE. Our previous study on wheat LTP1 localized a sequential B-cell epitope at position #24–33, comprising the cysteine doublet C27-C28. This epitope was detected with exceptionally high intensity compared with other bound LTP1 peptides^34^. In the case of Art v 3 (from mugwort pollen)^41^, C74 was evidenced as an epitope residue in direct interaction with antibodies.

Two other disulfide bonds, C3-C50 and C48-C87, link the H3 helix to the N- and C-terminal domains of the protein, respectively. Mutation of the C3-C50 bond did not change the secondary structure of wheat LTP1 and had almost no effect on IgE-binding or basophil triggering activity. Mutation of the C48-C87 bond only slightly modified LPT1 protein folding and antigenicity.

This difference in disulfide bond involvement and the essential role of C13-C27 and C28-C73 in LTP allergenicity has also been reported for other LTPs. Indeed, in Par j 1 (LTP from *Parietaria judaica* pollen)^48^, disruption of Cys14–Cys29 and Cys30–Cys75 bridging caused the loss of specific IgE-binding and of triggering activity for most patients, whereas a mutant lacking the other bridges retained its activity. The importance of the cysteine doublet in preserving LTP1 structure and allergenicity was also described for Pru p 3^33^. Apart from these two bridges, holding α-helices H1, H2 and H4 together might be an important structural element of LTP1 antigenicity.

In addition to residues C13, C27, C28 and C73, our data point to K72 as a key residue in IgE-binding and basophil triggering for all sera tested. SRCD results show that the K72A variant does not impact structure folding. This result points out that K72A may be directly involved in the interaction with the antibody via the relative protrusion of its lateral chain. Substitution of K72A by a small alanine residue induced changes in electrostatic properties, decreased protein volume and solvent accessible surface, and modified non-covalent interactions with residues from the H1 and H4 helices and from the loop between those helices (Fig. 6a). This substitution also had a dramatic effect on mAb recognition since it abolished binding for 7/8 anti-Nat-LTP mAbs, highlighting the essential involvement of K72 in LTP1 antigenicity. Residue K72 is well conserved among fruit and cereal LTP1s. Together with cysteine C73, it belongs to epitope zones already revealed on Tri a 14 (66–80) and on other allergenic LTPs, Pru p 3 (71–80)^35^ and Art v 3 (conformational epitope comprising K73 and C74)^41^. Its position between a proline that reduces the trypsin proteolytic activity and a cysteine forming a disulfide bridge may assure its protection from enzymatic digestion.

With respect to the 3D structure of LTP1, the five residues whose mutation greatly affected IgE-binding (C13, C27, C28, C73 and K72) cluster on the protein surface (Fig. 1d). The low variability in reactivity to the corresponding LTP1 variants as a function of the sera indicates that these residues may be included in dominant epitope regions.

Other mutations aimed to change electrostatic potential, volume or distance on LTP1 portions 39–44 or N76-L77 that belong to sequential epitopes (37–46 and 66–80) previously identified in food allergy to wheat or baker’s asthma^34,35^. These residues are localized in flexible parts of the protein (loop and the C-terminal region). As expected, we observed a visible influence of these single and double mutations in the SRCD spectra of the protein or variations in the content of the secondary structure. Despite these structural modifications, weak reductions in LTP1 antigenicity were detected in only two of these variants (D43N, R39A-D43N) and only for a limited number of sera. Other studies on Pru p 3 also observed a large effect of similar mutations only when they were in combination (e.g. simultaneous mutations of 39–40–43–44–76–77–78–79)^38^. Similarly, N77 appeared in a conformational epitope area of Art v 3 in combination with other residues (24, 33, 34, 36 and 39)^41^.

Additional characterization of the modifications induced by some mutations (C3S-C50S, C13S-C27S, C48S-C87S, D43N, R39A-D43N and K72A) on LTP1 structure and antigenicity was carried out with mouse mAbs directed against natural and denatured LTP1. Like patient sera, none of the anti-Nat-LTP mAbs recognized Ra-LTP. About half (8/14) of them equally bound to Nat-LTP and Wt-LTP. Very interestingly, these mAbs react similarly to patient sera towards the set of tested LTP1 variants, indicating that they surely bind the dominant IgE epitope regions containing the cluster C13, C27, C28, C73 and K72. This was also the case of mAbs generated to Art v 3, which showed good overlap with patient IgE^41^. This sub-group of anti-Nat-LTP mAbs could enable generation of recombinant monoclonal IgE as substitutes mimicking patients reactivity^49^.

In contrast to epitopes bound by anti-Nat-LTP, immunization with Ra-LTP mainly generated mAbs directed to new epitope sites that were moderately or not accessible in Nat-LTP or Wt-LTP but more accessible in the cysteine variants. Even with no or little modification observed in SRCD, binding of cysteine variants (C3-C50, C13-27, C48-C87) by anti-Ra-LTP mAbs indicated some change in the 3D structure of wheat LTP1 and in accessibility to some of these mAb epitopes. However, high-resolution 3D studies would be needed to provide more information about structural differences.

The reactivity of the panel of mAbs generated by immunization with purified Nat-LTP or Ra-LTP indicated small differences in the antigenicity of Nat-LTP and Wt-LTP. However, because most patient sera reacted equally to Nat-LTP and Wt-LTP in ELISA, the human polyclonal IgE repertoire appeared mostly restricted to regions with similar structural features or that may be less sensitive than mAbs to structural variations between the two proteins.

In conclusion, the similar reactivity of some mAbs and of IgE from allergic patients highlights the significant involvement of some residues in wheat LTP1 antigenicity.

We observed for the first time a cluster of residues (C13, C27, C28, C73 and K72) that appear essential for IgE-binding on wheat LTP1 or for IgE cross-linking on the FcεRI receptor. Among other properties, these residues play a definite role in the 3D structure of the antigenic site for its maintenance (disulfide bonds) or in terms of protrusion (K72). Spatial clustering and steric orientation of epitopes have been reported in food and respiratory allergens^50–52^ and proposed as a factor in facilitating allergenic activity. However, this cluster might contain several overlapping or non-overlapping epitopes^53^. Our work also highlights the hierarchy of disulfide bridges and the special participation of C13-C27 and C28-C73 in maintaining protein structure and allergenic potency. This result, also observed for LTPs in peach and *P. judaica* pollen, could eventually prove generic to numerous allergenic LTPs. Aside from these critical elements, we observed a moderate role of residues D43, R39, C48 and C87 in LTP1 antigenicity.

The three variants C13S-C27S, C28S-C73S and K72A appear to be promising hypoallergenic components. The efficacy of such protein variants as candidates usable in allergy immunotherapy remains to be investigated. In particular, their reduced triggering capacity as well as their immunogenicity should be evaluated on a larger sample of patient sera. In particular, the C13S-C27S and K72A variants, which folded quite similarly to Wt-LTP, should be tested for their capacity to modulate an allergic response to wheat LTP1.

## Methods

### Human subjects

Serum samples (*n* = 27) from patients diagnosed with food allergy to wheat were selected based on their sensitization to wheat LTP1 established by ELISA (Table S1). These sera were from 24 children and 3 adults presenting atopic eczema dermatitis syndrome, anaphylaxis, exercise-induced anaphylaxis, asthma, urticaria or gastrointestinal disorder. Sensitization to wheat proteins was assessed in patients using skin prick tests (SPTs) to wheat flour (Moulins Soufflet), gluten (ALK-Abelló) and to natural wheat LTP1 and/or using specific IgE assays to wheat flour or gluten (Phadia ImmunoCAP), total gliadin and albumin/globulin extracts (ELISA). A wheat allergy was confirmed by a positive challenge or evident effect of wheat avoidance as described^34^. Blood collection, SPT and challenges were performed with the informed consent of the patients or their parents and after receiving biomedical research approval from the Ethics Committee of Ile de France III and AFSSAPS (French Health Products Safety Agency, authorization no. 2008-A01565-50). All methods were performed in accordance with the relevant guidelines and regulations.

### Site-directed mutagenesis

The pETDP*ltp* expression vector enabling production of recombinant wild-type wheat LTP1 (UniProt P24296) as a fusion protein (Wt-LTP) with Trx was kindly provided by Dr K. Elmorjani (INRAE-BIA, Nantes, France) and used as a template to perform site-directed mutagenesis by a polymerase chain reaction (PCR)-based strategy. The wheat LTP1 coding sequence was inserted into the pET32-b expression vector (Novagen, San Diego, USA) between NcoI and HindIII restriction sites. cDNA was inserted as translational fusion protein with N-terminal Trx separated by a 50-residue-long spacer as previously reported^42^. The spacer contained six His-tag peptides for purification on a Ni^2+^ affinity column.

### Protein production and purification

Nat-LTP was purified as described by Douliez et al.^54^. Ra-LTP was obtained as follows: Nat-LTP was incubated with 0.1 M dithiothreitol (Merck, Saint-Quentin-Fallavier, France) for 2 h at 37 °C, then with 0.5 M iodoacetamide (Merck) in 0.5 M NaOH for 30 min in the dark. Afterwards, the protein solutions were dialysed overnight at room temperature, with change of dialysis bath every 8 h. Recombinant expression was done in *Escherichia coli* Origami strain (Novagen) as described in Elmorjani et al.^42^, except that recombinant LTP1 was not cleaved from Trx. After cell lysis by sonication, the soluble fraction (soluble proteins in 20 mM Tris–HCl pH 8, 200 mM NaCl, 20 mM imidazole) was applied on a HisTrap FF column (GE Healthcare, Merck, France) for Ni^2+^ affinity chromatography. Elution was performed with a 50–300 mM imidazole gradient. This first purification step was followed by anion exchange chromatography with Resource Q resin (GE Healthcare, Merck) on an Äkta Avant system (Cytiva, Fisher Scientific, Illkirch, France). The column was equilibrated with Tris 20 mM pH 8 before injection of the protein solution, and elution was performed by a NaCl gradient (up to 1 M) in Tris–HCl 20 mM pH 8. The proteins were extensively dialysed against water then lyophilized. The purity was assessed by SDS-PAGE, and the protein concentration was assayed with the BCA reagent kit (Pierce, Thermo Fisher, Illkirch, France) protein assay using BSA as standard. Protein identity was checked by mass spectrometry analysis (BIBS platform, INRA, Nantes, France). Recombinant Trx from *E. coli* (GenBank accession no. M26133) used as a control for IgE reactivity was purchased from Merck (#T0910 reference).

### Computational exploration of wheat ns-LTP1 structure

Visualization and manipulation of LTP1 3D structure were done using PyMOL software (https://pymol.org). Solvent accessibility area (or surface) (SASA) was calculated using the web server^55^. Sequence conservation was explored by multiple sequence alignment performed by Jalview^56^ and Clustal Omega (https://www.ebi.ac.uk/Tools/msa/clustalo/). Sequences were collected from the UniProt database (https://www.uniprot.org/) and queried on the keyword “NLTP1”. The DynaMut web tool (http://biosig.unimelb.edu.au/dynamut/)^43^ was used to predict the mutation effect on protein thermodynamic properties using the 1GH1 PDB file as a template. The impact of mutation on volume and surface properties was computed using the VLDP and protein volume web servers (VLDP: https://www.dsimb.inserm.fr/dsimb_tools/vldp/)^44^ using the wild-type or mutated protein generated by DynaMut as templates. Finally, wild-type or mutated protein 3D models were used as templates to predict protein electrostatic properties using PyMol.

### Production of mAbs to the Nat-LTP1 and Ra-LTP

Two groups of three BALB/c mice were injected subcutaneously in the rear legs, twice, at 2-week intervals, with 20 µg of Nat-LTP (group 1) or Ra-LTP (group 2) emulsified in TiterMax Gold Adjuvant (CytRx Corporation, Merck). Three days after the second immunization, the mice were sacrificed. For each group, the popliteal lymph nodes were removed, and their lymphocytes were suspended by perfusion, pooled and and fused with the SP2/0 myeloma cell line using standard protocol for hybridoma preparation. Hybridoma supernatants from the fusion were screened for the presence of anti-immunogen antibodies using an indirect ELISA. Nat-LTP or Ra-LTP were coated overnight at 5 µg/mL in PBS on MaxiSorp ELISA plates (Thermo Fisher, Nunc, Illkirch, France). After three washes in PBS-0.05% Tween 20, wells were saturated with 4% skimmed milk powder in PBS for 2 h at room temperature. After three washes in PBS-0.05% Tween 20, hybridoma supernatants were incubated for 1 h at room temperature. After three washes in PBS-Tween 0.05%, mAbs bound to antigen were detected with peroxidase-conjugated goat anti-mouse IgG (H + L) (Bio-Rad, Marnes-la-Coquette, France) and revealed with ortho-phenylenediamine (Merck). Selected hybridomas were cloned by dilution. Animal experiments were carried out at the INRAE facilities, which are authorized by the local veterinary department (authorization no. 44502). All animal experiments were approved by the Ethics Committee of the Pays de la Loire (registration no. CEEA-2011.26). The study was carried out, where appropriate, in compliance with the ARRIVE guidelines.

### Reactivity of mAbs to LTP1s

The reactivity of mAbs directed to either Nat-LTP or Ra-LTP to Mut-LTP, Nat-LTP and Ra-LTP was assessed by indirect ELISA as described above, using four dilutions of hybridoma supernatant (1/2, 1/20, 1/200, 1/2000). For each mAb, titration curves were built and the highest dilution of the supernatant giving a signal at the plateau of the curve was determined for each Mut-LTP, Nat-LTP or Ra-LTP. When no reactivity was observed, the reported dilution was set at zero for the next calculation. For Nat-LTP mAbs, we calculated the ratio of the dilution determined for either Mut-LTP or for Ra-LTP to the dilution determined for Nat-LTP. For Ra-LTP mAbs, we calculated the ratio of the dilution determined for each Mut-LTP or for Nat-LTP to the dilution determined for Ra-LTP. Reactivity of a mAb to each LTP1 variant form was considered equivalent to that observed on the immunogen (Nat-LTP or Ra-LTP) if the ratio was superior to 0.1, reduced if the ratio was between 0.01 and 0.1, and absent if the ratio was inferior to 0.01.

### Measurement of IgE-binding to Nat-LTP1 and recombinant Wt-LTP and Mut-LTPs in F-ELISA

LTP- and Trx- specific IgE concentrations were measured by F-ELISA as described in Mameri et al*.*^27^. Nat-LTP, Ra-LTP, or Trx (Merck) solubilized in PBS buffer were coated at 5 µg mL^–1^ in carbonate buffer, and sera were diluted 1:10 or 1:40. All determinations were carried out in triplicate. A range of standard IgEs (human serum immunoglobulin E: NIBSC ref. 75/502) from 0.8 ng mL^–1^ up to 160 ng mL^–1^ were processed by sandwich ELISA, using rabbit anti-human IgE (Dako France S.A.S., Trappes, France) as capture antibody. The specific IgE concentrations in the sera were calculated from the fluorescent signal and the standard curve after adjusting a four-parameter logistic regression curve (GraphPad Software) to the data.

### RBL cell degranulation assay

Activation of rat basophil leukaemia RBL cells was carried out with the SX38 P4B7 clone expressing the human FcεRI α-, β- and γ-chains as described^27,57^. Proteins (Nat-LTP, Ra-LTP, Wt-LTP and seven Mut-LTP1s) were tested at six concentrations (0.01, 0.1, 1, 10, and 100 and 1000 ng mL^–1^) with six sera diluted to 1:10 (except #1639, which was diluted 1:50). Sera were selected based on availability and IgE concentrations specific for Nat-LTP and Wt-LTP. We calculated EC_50_ for each serum and protein, as the protein concentration allowing 50% of the degranulation maximum.

### SRCD

SRCD spectra were recorded as previously described in Vindigni et al*.*^58^ and Gohon et al*.*^59^ on the DISCO beamline at the SOLEIL synchrotron facility (Gif-sur-Yvette, France). Briefly, proteins were buffer exchanged with 10 mM phosphate buffer, pH 8, containing 100 mM Na_2_SO_4_. CD spectra recorded at 25 °C at wavelengths between 170 and 280 nm with a data pitch of 1 nm were measured at least three times for each protein sample and its buffer counterparts. After subtraction of averaged buffer spectra from those of samples, the 263–270 nm region was set to zero. The resulting spectra were calibrated with d-10-camphorsulfonic acid using CDtoolX software^60^. Spectra were normalized to protein concentrations and optical path length and presented in Δε units. Spectral deconvolution, fitting and secondary structure percentages were calculated using the publicly available BeStSel web server^61^. Spectra were analysed according to wavelength range quality and accuracy validity: this corresponds to the photomultiplier high tension remaining below half of its total variation. Normalized root-mean-square deviations provided the most accurate fit for each spectrum. Value ranged from 0.01 to 0.08.

### Statistical analyses

Statistical analyses were performed using GraphPad Prism, version 9.1.2 for Windows (GraphPad Software, San Diego, CA, USA) with the significance level set at 0.05. IgE reactivity towards Nat- and Wt-LTP and Trx was compared using ANOVA (Dunn’s multiple comparisons test), while a Wilcoxon test was applied to compare IgE reactivity towards Nat-LTP and variants, as these data often failed to demonstrate a Gaussian distribution (d’Agostino–Pearson omnibus normality test).

## Supplementary Information

Supplementary Legends.

Supplementary Figure S1.

Supplementary Figure S2.

Supplementary Table S1.

## Data Availability

The other datasets used and/or analysed during the current study available from the corresponding author on reasonable request.

## References

[1] Kader J-C (1996). Lipid-transfer proteins in plants. Annu. Rev. Plant Physiol. Plant Mol. Biol..

[2] Skypala IJ (2021). Non-specific lipid-transfer proteins: Allergen structure and function, cross-reactivity, sensitization, and epidemiology. Clin. Transl. Allergy.

[3] Wong LH, Čopič A, Levine TP (2017). Advances on the transfer of lipids by lipid transfer proteins. Trends Biochem. Sci..

[4] Wong LH, Gatta AT, Levine TP (2019). Lipid transfer proteins: The lipid commute via shuttles, bridges and tubes. Nat. Rev. Mol. Cell Biol..

[5] Gonzalez-Klein Z (2021). The key to the allergenicity of lipid transfer protein (LTP) ligands: A structural characterization. Biochim. Biophys. Acta BBA Mol. Cell Biol. Lipids.

[6] Breiteneder H, Radauer C (2004). A classification of plant food allergens. J. Allergy Clin. Immunol..

[7] Incorvaia C (2014). Food allergy as defined by component resolved diagnosis. Recent Pat. Inflamm. Allergy Drug Discov..

[8] Kalla R (1994). The promoter of the barley aleurone-specific gene encoding a putative 7 kDa lipid transfer protein confers aleurone cell-specific expression in transgenic rice. Plant J..

[9] Marion D, Bakan B, Elmorjani K (2007). Plant lipid binding proteins: Properties and applications. Biotechnol. Adv..

[10] Gincel E (1994). Three-dimensional structure in solution of a wheat lipid-transfer protein from multidimensional 1H-NMR data. A new folding for lipid carriers. Eur. J. Biochem..

[11] Charvolin D, Douliez JP, Marion D, Cohen-Addad C, Pebay-Peyroula E (1999). The crystal structure of a wheat nonspecific lipid transfer protein (ns-LTP1) complexed with two molecules of phospholipid at 2.1 A resolution. Eur. J. Biochem..

[12] Pastorello EA (2002). Hypersensitivity to mugwort (*Artemisia vulgaris*) in patients with peach allergy is due to a common lipid transfer protein allergen and is often without clinical expression. J. Allergy Clin. Immunol..

[13] Asero R, Pravettoni V (2013). Anaphylaxis to plant-foods and pollen allergens in patients with lipid transfer protein syndrome. Curr. Opin. Allergy Clin. Immunol..

[14] Pastorello EA, Robino AM (2004). Clinical role of lipid transfer proteins in food allergy. Mol. Nutr. Food Res..

[15] Salcedo G, Sanchez-Monge R, Diaz-Perales A, Garcia-Casado G, Barber D (2004). Plant non-specific lipid transfer proteins as food and pollen allergens. Clin. Exp. Allergy.

[16] Lauer I (2007). Identification of a plane pollen lipid transfer protein (Pla a 3) and its immunological relation to the peach lipid-transfer protein, Pru p 3. Clin. Exp. Allergy.

[17] Palacín A (2012). Graph based study of allergen cross-reactivity of plant lipid transfer proteins (LTPs) using microarray in a multicenter study. PLoS ONE.

[18] Pascal M (2012). Lipid transfer protein syndrome: Clinical pattern, cofactor effect and profile of molecular sensitization to plant-foods and pollens. Clin. Exp. Allergy.

[19] Pastorello EA (2013). Anti-rPru p 3 IgE levels are inversely related to the age at onset of peach-induced severe symptoms reported by peach-allergic adults. Int. Arch. Allergy Immunol..

[20] Palacin A (2007). Wheat lipid transfer protein is a major allergen associated with baker’s asthma. J. Allergy Clin. Immunol..

[21] Pastorello EA (2000). The maize major allergen, which is responsible for food-induced allergic reactions, is a lipid transfer protein. J. Allergy Clin. Immunol..

[22] Pastorello EA (2014). Wheat-dependent exercise-induced anaphylaxis caused by a lipid transfer protein and not by ω-5 gliadin. Ann. Allergy Asthma Immunol..

[23] Battais F (2005). Food allergy to wheat: Differences in IgE-binding proteins as a function of age or symptoms. J. Cereal Sci..

[24] Pastorello EA (2007). Wheat IgE-mediated food allergy in European patients: Alpha-amylase inhibitors, lipid transfer proteins and low-molecular-weight glutenins—allergenic molecules recognized by double-blind, placebo-controlled food challenge. Int. Arch. Allergy Immunol..

[25] Mäkelä MJ (2014). Wheat allergy in children—new tools for diagnostics. Clin. Exp. Allergy.

[26] Sander I (2011). Multiple wheat flour allergens and cross-reactive carbohydrate determinants bind IgE in baker’s asthma: Recombinant wheat flour allergens in baker’s asthma. Allergy.

[27] Mameri H (2012). Molecular and immunological characterization of wheat Serpin (Tri a 33). Mol. Nutr. Food. Res..

[28] Gómez-Casado C (2014). Component-resolved diagnosis of wheat flour allergy in baker’s asthma. Allergy Clin. Immunol..

[29] Zuidmeer-Jongejan L (2012). FAST: Towards safe and effective subcutaneous immunotherapy of persistent life-threatening food allergies. Clin. Transl. Allergy.

[30] Gómez-Casado C (2013). Allergenic characterization of new nutant forms of Pru p 3 as new immunotherapy vaccines. Clin. Dev. Immunol..

[31] Toda M (2011). Protein unfolding strongly modulates the allergenicity and immunogenicity of Pru p 3, the major peach allergen. J. Allergy Clin. Immunol..

[32] Rodriguez MJ (2017). Pru p 3-epitope-based sublingual immunotherapy in a murine model for the treatment of peach allergy. Mol. Nutr. Food Res..

[33] Eichhorn S (2019). Rational design, structure–activity relationship, and immunogenicity of hypoallergenic Pru p 3 variants. Mol. Nutr. Food Res..

[34] Denery-Papini S (2011). Immunoglobulin-E-binding epitopes of wheat allergens in patients with food allergy to wheat and in mice experimentally sensitized to wheat proteins. Clin. Exp. Allergy.

[35] Tordesillas L (2009). Molecular basis of allergen cross-reactivity: Non-specific lipid transfer proteins from wheat flour and peach fruit as models. Mol. Immunol..

[36] Mamone G (2015). Tracking the fate of pasta (*T. durum* Semolina) immunogenic proteins by in vitro simulated digestion. J. Agric. Food Chem..

[37] Abdullah SU (2016). Ligand binding to an allergenic lipid transfer protein enhances conformational flexibility resulting in an increase in susceptibility to gastroduodenal proteolysis. Sci. Rep..

[38] Garcia-Casado G (2003). Identification of IgE-binding epitopes of the major peach allergen Pru p 3. J. Allergy Clin. Immunol..

[39] Pacios LF (2008). Mimotope mapping as a complementary strategy to define allergen IgE-epitopes: Peach Pru p 3 allergen as a model. Mol. Immunol..

[40] Tordesillas L (2009). T-cell epitopes of the major peach allergen, Pru p 3: Identification and differential T-cell response of peach-allergic and non-allergic subjects. Mol. Immunol..

[41] Di Muzio M (2020). Hydrogen/deuterium exchange memory NMR reveals structural epitopes involved in IgE cross-reactivity of allergenic lipid transfer proteins. J. Biol. Chem..

[42] Elmorjani K, Lurquin V, Lelion A, Rogniaux H, Marion D (2004). A bacterial expression system revisited for the recombinant production of cystine-rich plant lipid transfer proteins. Biochem. Biophys. Res. Commun..

[43] Rodrigues CH, Pires DE, Ascher DB (2018). DynaMut: Predicting the impact of mutations on protein conformation, flexibility and stability. Nucleic Acids Res..

[44] Esque J, Léonard S, de Brevern AG, Oguey C (2013). VLDP web server: A powerful geometric tool for analysing protein structures in their environment. Nucleic Acids Res..

[45] Mueller GA (2011). Ara h 2: Crystal structure and IgE binding distinguish two subpopulations of peanut allergic patients by epitope diversity. Allergy.

[46] Dubiela P (2017). Enhanced Pru p 3 IgE-binding activity by selective free fatty acid-interaction. J. Allergy Clin. Immunol..

[47] Aina R (2019). Distinct lipid transfer proteins display different IgE-binding activities that are affected by fatty acid binding. Allergy.

[48] Bonura A (2001). Hypoallergenic variants of the *Parietaria judaica* major allergen Par j 1: A member of the non-specific lipid transfer protein plant family. Int. Arch. Allergy Immunol..

[49] Tranquet O (2017). A chimeric IgE that mimics IgE from patients allergic to acid-hydrolyzed wheat proteins is a novel tool for in vitro allergenicity assessment of functionalized glutens. PLoS ONE.

[50] Flicker S (2006). Spatial clustering of the IgE epitopes on the major timothy grass pollen allergen Phl p 1: Importance for allergenic activity. J. Allergy Clin. Immunol..

[51] Gieras A (2016). IgE epitope proximity determines immune complex shape and effector cell activation capacity. J. Allergy Clin. Immunol..

[52] Hochwallner H (2010). Visualization of clustered IgE epitopes on α-lactalbumin. J. Allergy Clin. Immunol..

[53] Van Regenmortel MHV (2011). Limitations to the structure-based design of HIV-1 vaccine immunogens. J. Mol. Recogn..

[54] Douliez JP (2001). Identification of a new form of lipid transfer protein (LTP1) in wheat seeds. J. Agric. Food Chem..

[55] Fraczkiewicz R, Braun W (1998). Exact and efficient analytical calculation of the accessible surface areas and their gradients for macromolecules. J. Comput. Chem..

[56] Waterhouse AM, Procter JB, Martin DMA, Clamp M, Barton GJ (2009). Jalview Version 2—A multiple sequence alignment editor and analysis workbench. Bioinformatics.

[57] Wiegand TW (1996). High-affinity oligonucleotide ligands to human IgE inhibit binding to Fc epsilon receptor I. J. Immunol..

[58] Vindigni J-D (2013). Fold of an oleosin targeted to cellular oil bodies. Biochim. Biophys. Acta BBA Biomembr..

[59] Gohon Y (2011). High water solubility and fold in amphipols of proteins with large hydrophobic regions: Oleosins and caleosin from seed lipid bodies. Biochim. Biophys. Acta BBA Biomembr..

[60] Miles AJ, Wallace BA (2018). CDtoolX, a downloadable software package for processing and analyses of circular dichroism spectroscopic data. Protein Sci..

[61] Micsonai A (2018). BeStSel: A web server for accurate protein secondary structure prediction and fold recognition from the circular dichroism spectra. Nucleic Acids Res..

