# Supplementary material for: Critical structural elements for the antigenicity of wheat allergen LTP1 (Tri a 14) revealed by site-directed mutagenesis
Source: Sci Rep. 2022 Jul 18;12:12253. doi: 10.1038/s41598-022-15811-5 (PMC9293932; doi:10.1038/s41598-022-15811-5)
Supplement: Supplementary file 1 — Supplementary Legends. [file 41598_2022_15811_MOESM1_ESM.docx]

**Supplementary figures:**

**Table S1: Patient clinical data.**

Clinical data for patients with food allergy to wheat used in the present study.

**Figure S1:** **a.** Multiple sequence alignment of an extended set of 23 plant LTP1 sequences (lipid transfer proteins) generated using Jalview Software^56^. **b.** Scatterplot showing the relative surface accessibility of LTP1 residues computed on 1GH1 PDB structure. The amino acids targeted by mutagenesis are indicated with colours (cysteine residues are in red; other amino acids are in other colours).

**Figure S2: LTP1 SRCD spectra.**

**a.** SRCD spectra and secondary structure content estimation of native LTP1 (Nat-LTP), reduced-alkylated LTP1 (Ra-LTP), recombinant wild-type wheat LTP1 (Wt-LTP) and recombinant thioredoxin (Trx). **b.** Bar plot of the secondary structure content of Nat-LTP, Ra-LTP, Wt-LTP and Trx estimated by the BeStSel algorithm. To guide the reader, the SRCD signal of Wt-LTP1 is the same as that presented in Fig. 5. LTP1: lipid transfer protein. SRCD: synchrotron radiation circular dichroism.
