# Supplementary material for: Critical structural elements for the antigenicity of wheat allergen LTP1 (Tri a 14) revealed by site-directed mutagenesis
Source: Sci Rep. 2022 Jul 18;12:12253. doi: 10.1038/s41598-022-15811-5 (PMC9293932; doi:10.1038/s41598-022-15811-5)
Supplement: Supplementary file 2 — Supplementary Figure S1. [file 41598_2022_15811_MOESM2_ESM.pdf]

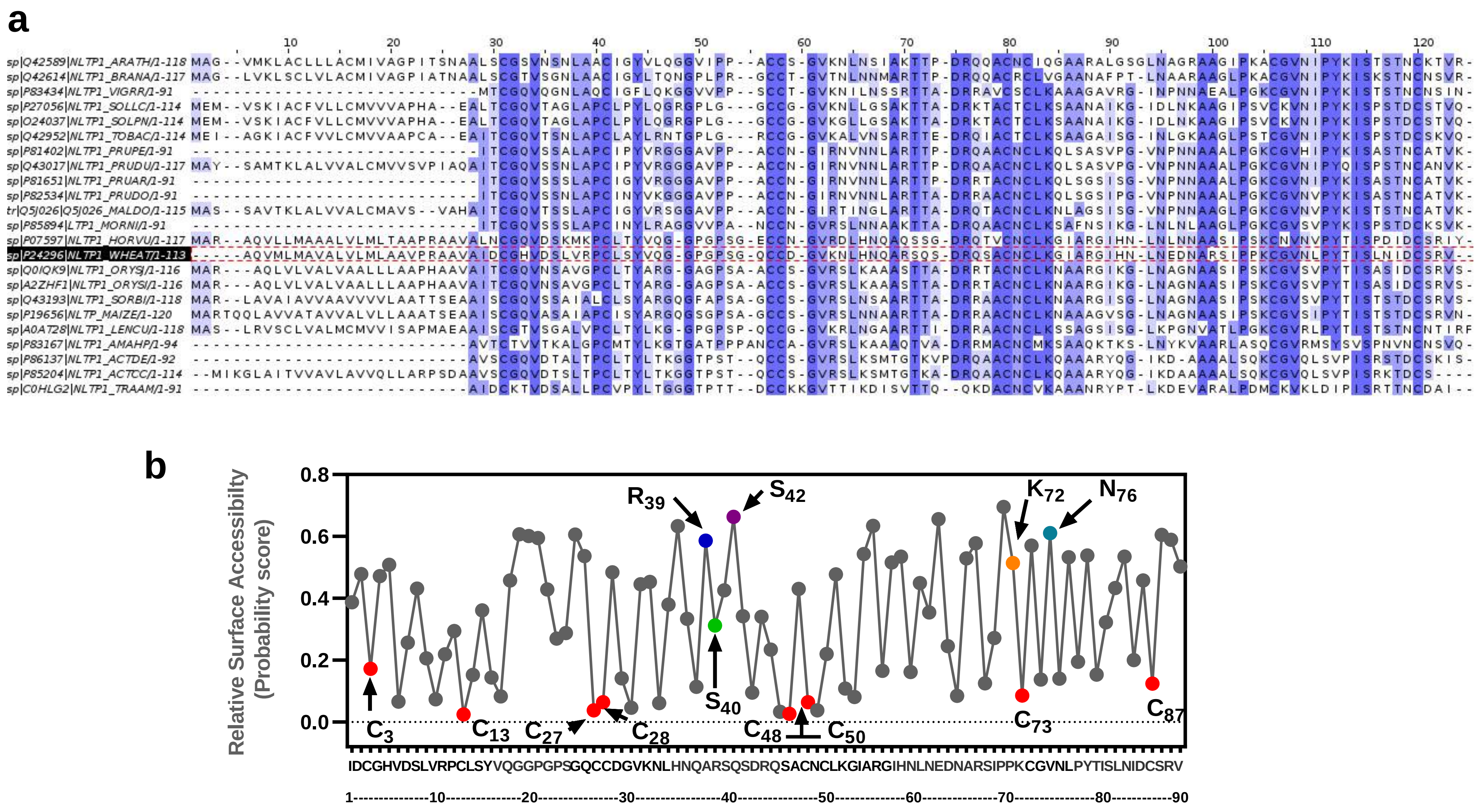

a
b
Relative Surface Accessibility
(Probability score)
0.8
0.6
0.4
0.2
0.0
R39
S42
K72
N76
S40
C3
C13
C27
C28
C48
C50
C73
C87
IDCGHVDSLVRPCLSYVQGGPGPSGQCCDGVKNLHNQARSQSDRQSACNCLKGIARGIHNLNEDNARSIPPKCGVNLPYTISLNIDCSRV
1--------------10--------------20--------------30--------------40--------------50--------------60--------------70--------------80------------90
