# Supplementary figures and images for: Critical structural elements for the antigenicity of wheat allergen LTP1 (Tri a 14) revealed by site-directed mutagenesis
Source: Sci Rep. 2022 Jul 18;12:12253. doi: 10.1038/s41598-022-15811-5 (PMC9293932; doi:10.1038/s41598-022-15811-5)

Figure S2

a

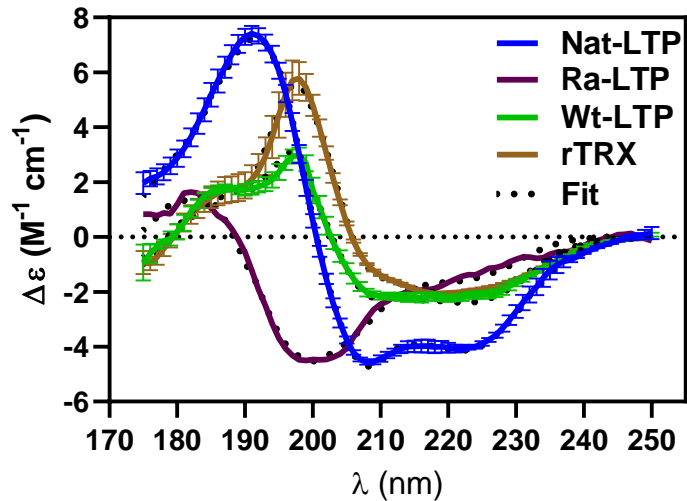

b

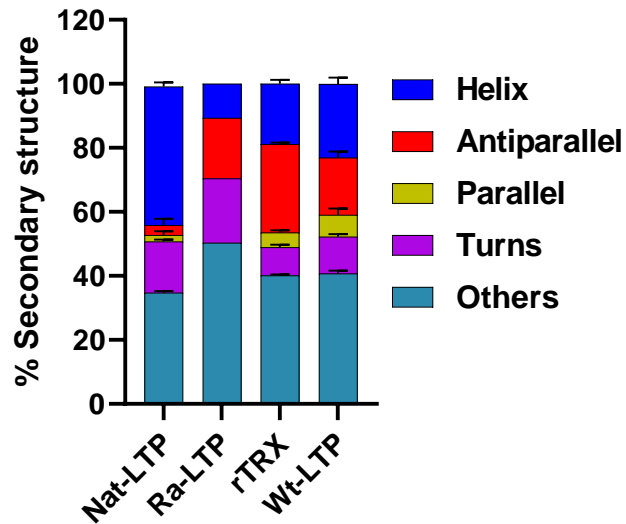

Supplement: Supplementary file 3 — Supplementary Figure S2. [file 41598_2022_15811_MOESM3_ESM.pdf]
