# Supplementary material for: Critical structural elements for the antigenicity of wheat allergen LTP1 (Tri a 14) revealed by site-directed mutagenesis
Source: Sci Rep. 2022 Jul 18;12:12253. doi: 10.1038/s41598-022-15811-5 (PMC9293932; doi:10.1038/s41598-022-15811-5)
Supplement: Supplementary file 4 — Supplementary Table S1. [file 41598_2022_15811_MOESM4_ESM.docx]

|  |  | | |  |  | **Skin prick test (mm)** | | | **Specific IgE in CAP KUI/L** | | **Total IgE in ELISA ng/ml** | **Specific IgE in ELISA ng/ml** | | | **Food**  **challenge**  Reactive dose |  |
| --- | --- | --- | --- | --- | --- | --- | --- | --- | --- | --- | --- | --- | --- | --- | --- | --- |
| **Sera** | **Sex/age** | | **Symptoms** | | | Wheat | Gluten | LTP1 | Wheat | Gluten |  | LTP1 | Gliadins | A/G |  |  |
| 1 | M | Child | AEDS, Asth | | | 4 |  |  |  |  |  | 99 |  | 30 | Pos 10 g flour | |
| 2 | F | 2 | AEDS, Asth, U | | | 4 | 5 |  | 60 | 27 | 3000 | 223 | 16 | 15 | Pos 5 g flour | |
| 3 | F | 3 | AEDS | | | 5 | 4 |  | >100 | 0 | 12830 | 49 | 40 | 102 | Pos 5 g flour | |
| 4 | M | Child |  | | |  |  |  | 76 | 64 | 11977 | 78 |  |  |  | |
| 5 | F | 6 | AEDS, Asth, U | | | 6 | Pos |  | 40 | 25 | 809 | 136 | 29 | 171 | Pos 5 g flour | |
| 6 | F | 1 | AEDS, U | | |  |  |  |  |  | 327 | 14 | 0 | 0 | Nd* | |
| 7 |  | E | AEDS | | |  |  |  |  |  | 1130 | 23 | 5 | 57 |  | |
| 8 | F | 9 | AEDS | | | 0 |  |  |  |  | 4150 | 119 |  | 23 | Pos 70 g flour | |
| 9 | M | 4 | AEDS | | | Pos | Pos |  |  | Pos | 262 | 28 | 10 | 26 |  | |
| 10 | F | 5 | AEDS | | | 9.5 |  |  |  |  | 524 | 76 | 178 | 154 | LT | |
| 11 | M | 18 | EIA | | | 3.5 |  | 24 | 4.8 | 1.3 | 877 | 21 | 0 | 14 | Neg * | |
| 12 | M | 5 | AEDS | | | 3 |  | 14 | 100 | 100 | 6677 | 118 | 124 | 86 |  | |
| 13 | M | 5 | GI | | | 5 | 0 |  | 8.9 | 0 | 1076 | 27 | 0 | 12 | Neg 4.5g flour* | |
| 14 | M | 9 | GI | | | 3.5 | 3 | 5 | 100 | 71 | 5816 | 135 | 101 | 60 | Pos 10 g flour | |
| 15 | F | 6 | AEDS, Asth | | | 15 | 6 | 17 | 100 | 100 | 8466 | 473 | 71 | 125 | Pos 965 mg flour | |
| 16 | F | 36 | U | | | 4 | 1 | 5 |  |  | 1087 | 67 | 0 | 0 | Pos | |
| 17 | M | 5 | AEDS, Asth | | | 8 | 8 | 16 | 44 | 36 | 710 | 64 | 46 | 74 | Pos 7 g flour | |
| 18 | M | 3 | AEDS | | | 3 | 3 |  | 2.5 | 0 | 1935 | 45 | 8 | 16 |  | |
| 19 | M | 4 | U | | | 5 | 6 |  |  | 36 | 2631 | 36 | pos | 17 |  | |
| 20 | M | 12 | EE0 | | | 8 | 4 |  |  |  | 9631 | 429 | 109 | 201 |  | |
| 21 | M | 9 | AS | | | 6 | 7 |  | 60.2 | 73.2 | 1476 | 156 | 75 | 143 | Nd* | |
| 22 | M | 8 | AS | | | 7 | 8 |  | 42.5 | 43.2 | 3945 | 245 | 80 | 86 | Pos 1 g flour | |
| 23 | M | 10 | U | | | 8 | 7 |  | 10 | 12.8 | 2213 | 74 | 7 | 19 |  | |
| 24 | M | 6 | Asth | | |  |  |  |  |  | 708 | 13 | 101 | 21 | Pos 4 g bread crust | |
| 25 | F | 36 | AS | | |  |  |  |  |  | 2348 | 29 | 0 | 66 |  | |
| 26 | F | 9 | AS | | | 9 | 10 |  | 100 | 100 | 1103 | 66 | 208 | 206 | Nd* | |
| 27 | M | 10 | U, Asth | | | 5 | 6 |  | 100 | 100 | 32390 | 955 | 7725 | 5641 | Nd* | |

Nd*: Not done, Positive effect of avoidance diet.

AEDS: atopic eczema dermatitis syndrome. AS: anaphylaxis. Asth : asthma. EEO: eosinophilic esophagitis. EIA: exercise induced anaphylaxis. ELISA: enzyme-linked immunosorbent assay. GI: gastrointestinal symptoms. IgE: immunoglobulin E. LT: labial test. U: urticaria.
